# Intestinal toxicity evaluation of TiO_2_ degraded surface-treated nanoparticles: a combined physico-chemical and toxicogenomics approach in caco-2 cells

**DOI:** 10.1186/1743-8977-9-18

**Published:** 2012-05-31

**Authors:** Matthieu Fisichella, Frederic Berenguer, Gerard Steinmetz, Melanie Auffan, Jerome Rose, Odette Prat

**Affiliations:** 1 CEA, IBEB, SBTN, LEPC, F-30207, Bagnols-sur-Cèze, France; 2 CEREGE, UMR 6635 CNRS/Aix-Marseille Université´,ECCOREV, Europôle de l’Arbois, F-13545, Aix-en-Provence, France; 3 International Consortium for the Environmental Implications of Nanotechnology (iCEINT), Aix-en-Provence, France; 4 CEA, IBEB, SBTN, Laboratoire d’Etude des Protéines Cibles, F-30207, Bagnols-sur-Cèze, France

**Keywords:** Nanoparticles (NPs), Surface-treated nanoparticles (STNPs), Titanium dioxide, Toxicity, degradation of nanomaterials, Gene expression, Life cycle

## Abstract

**Background:**

Titanium dioxide (TiO_2_) nanoparticles (NPs) are widely used due to their specific properties, like UV filters in sunscreen. In that particular case TiO_2_ NPs are surface modified to avoid photocatalytic effects. These surface-treated nanoparticles (STNPs) spread in the environment and might release NPs as degradation residues. Indeed, degradation by the environment (exposure to UV, water and air contact …) will occur and could profoundly alter the physicochemical properties of STNPs such as chemistry, size, shape, surface structure and dispersion that are important parameters for toxicity. Although the toxicity of surface unmodified TiO_2_ NPs has been documented, nothing was done about degraded TiO_2_ STNPs which are the most likely to be encountered in environment. The superoxide production by aged STNPs suspensions was tested and compared to surface unmodified TiO_2_ NPs. We investigated the possible toxicity of commercialized STNPs, degraded by environmental conditions, on human intestinal epithelial cells. STNPs sizes and shape were characterized and viability tests were performed on Caco-2 cells exposed to STNPs. The exposed cells were imaged with SEM and STNPs internalization was researched by TEM. Gene expression microarray analyses were performed to look for potential changes in cellular functions.

**Results:**

The production of reactive oxygen species was detected with surface unmodified TiO_2_ NPs but not with STNPs or their residues. Through three different toxicity assays, the STNPs tested, which have a strong tendency to aggregate in complex media, showed no toxic effect in Caco-2 cells after exposures to STNPs up to 100 μg/mL over 4 h, 24 h and 72 h. The cell morphology remained intact, attested by SEM, and internalization of STNPs was not seen by TEM. Moreover gene expression analysis using pangenomic oligomicroarrays (4x 44000 genes) did not show any change versus unexposed cells after exposure to 10 μg/ mL, which is much higher than potential environmental concentrations.

**Conclusions:**

TiO_2_ STNPs, degraded or not, are not harmful to Caco-2 cells and are unlikely to penetrate the body via oral route. It is likely that the strong persistence of the aluminium hydroxide layer surrounding these nanoparticles protects the cells from a direct contact with the potentially phototoxic TiO_2_ core.

## Background

The use of nanoparticles (NPs) has significantly increased during the last decade in several areas such as computer science, chemistry, cosmetics and pharmaceuticals. There is an urgent need to verify their harmlessness for human health and the environment, because the potential ability of NPs to penetrate the cells and generate internal damage blocks acceptance of these new materials by the public [1]. But there is no universal NP to fit all cases, and each nanomaterial has to be treated individually with respect to health effects [2]. Moreover, most of the time, NPs are surface modified to be incorporated in final commercialized products. Titanium dioxide (TiO_2_) NPs are widely used owing to their specific properties, like UV filters in sunscreens, outdoor paints or photocatalytic in glass or cements. Mueller and Novack estimated the predicted environmental concentration of TiO_2_ NPs at between 0.7 and 16 μg/L [3]. The question arises whether these nanoparticles cross biological barriers: skin, lung, intestine, brain. Jin et al. showed TiO_2_ NPs toxicity to mouse fibroblast cells at high concentration [4]. In human fibroblast cells, TiO_2_ NPs induce loss of viability at concentrations above 1 mg/L [5], meaning a concentration 100 times higher than the predicted environmental concentration. In a real-world exposition, three TiO_2_ sunscreen formulations were tested on human skin by nuclear microscopy and were shown to be unable to cross the human skin barrier [6]. In lung cells, surface unmodified TiO_2_ NPs induce loss of viability and ROS production [7]. Nevertheless, most of these studies focused on skin contact and inhalation, while ingestion of NPs is a major route of exposure. A way to provide an in vitro simulation of exposure via the oral route is to use the Caco-2 cell culture. It is one of the most relevant in vitro models to study the differentiation and regulation of intestinal functions [8]. To our knowledge, only one study by Koeneman et al. has evaluated the toxicity of surface unmodified TiO_2_ NPs on intestinal Caco-2 cells [9]. These authors support the hypothesis that these TiO_2_ nanoparticles enter cells by transcytosis without disturbing membrane integrity or inducing cell death. But the concentrations used reach 1 g/L, which is largely above assumed environmental levels. Furthermore, most authors used surface unmodified NPs while most of the TiO_2_ NPs used in commercialized products are surface modified. In the case of sunscreens containing TiO_2_ NPs (usually 1–5% by weight), these NPs are covered by different layers such as Al(OH)_3_ to prevent the generation of Reactive Oxygen Species (ROS) at the TiO_2_ NP surface [10] and surrounded with some hydrophobic or hydrophilic organic layers to better disperse the surface-treated TiO_2_ NPs in the final products [11]. All these layers strongly modify TiO_2_ NPs effects on skin damage for instance [12]. In a non-direct exposure scenario (sunscreen surface-treated nanoparticles released in aquatic systems) TiO_2_ STNPs should spread degradation residues in the environment [13]. Indeed, environmental degradation (exposure to UV, water and air contact …) will occur and could alter the physicochemical properties of STNPs, such as surface chemistry, crystalline structure, dispersion state, and of course the concentration in contact with the organisms, that are important parameters for toxicity. Moreover they can interact with benthic fauna, where they may be internalized by grazer organisms and eaten by humans [14,15]. These changes due to environmental exposure could therefore profoundly alter the toxicity of STNPs. After ingestion, degradation in the stomach can also change the physical chemistry of STNPs and their toxic properties. Wang et al. described an increase in toxicity by CdSe quantum dots in intestinal cells after acidic treatment. This increase is explained by the degradation of the protective PEG surface layer [16].

In addition, environmental concentrations are likely to be low and current toxicological methods cannot unravel the mechanisms of action of toxicants at low doses. Conversely toxicogenomics is a methodology capable of detecting subtle changes in cell function at the level of gene expression, even at very low doses. Moreover the global nature of gene expression analysis also provides a wealth of information for networking genes modulated by a toxic substance. In particular, toxicogenomics is a powerful tool for monitoring disturbed cellular pathways (i.e. oxidative stress, apoptosis, hypoxia, etc) under the influence of toxicants. Furthermore, the fine sorting of genes with altered expression can help to discover biomarkers of effect. For example, we recently used toxicogenomics to relate for the first time the uranyl ions to a gene, and its associated protein involved in the ectopic mineralization of metals [17,18].

This current work aims to evaluate the toxicity of commercialized TiO_2_ STNPs (T-Lite™ SF from BASF, also known as T-Lite) widely used in sunscreens, compared with their degradation residues generated after exposure to UV light (T-Lite DL) or acidic medium (T-Lite DA). T-Lite SF is made of a rutile core surrounded by a thin Aluminum Hydroxide layer, and surface treated by polydimethylsiloxane polymer (PDMS). It was shown by Auffan et al. [19] that after contact with water, the TiO_2_ STNP becomes hydrophilic and forms aggregates in suspension due to the desorption and oxidation of the outer amphiphilic PDMS coating layer. However, the aluminium hydroxide layer persists at the surface of the TiO_2_ STNP. The physico-chemical behavior of these TiO_2_ STNP in suspension has been studied by Labille at al. [15] in terms of aggregation states and surface charge.

The T-Lite DL were aged according to the protocol previously published [15] to simulate an environmental degradation. The T-Lite DA were produced according to a protocol set by Wang et al. to mimic gastro-intestinal degradation [16]. We used the Caco-2 cell line as a model of human intestinal epithelium, which expresses spontaneous enterocytic differentiation at confluence [20,21]. The study of these STNPs, degradation residues of nanomaterials, is appropriate, because they are components of many consumer products and they have a protective layer likely to be degraded by the environment.

### Experimental design

In the present investigation, we use an integrated strategy to determine noxious effects of degraded surface-treated TiO_2_ NPs. Physicochemical properties (e.g. shape, size, aggregation state, zeta potential and crystal structure) have been previously reported [14,15] for T-Lite DL in water, but were analyzed in the present study, with the T-Lite DA, in the media used to expose the cell cultures. The generation of Reactive Oxygen Species (ROS) from the STNPs suspensions was also measured as a probe for surface modification. We looked for toxic concentrations of these characterized STNPs for Caco-2 cells, using several cytotoxicity tests (Trypan Blue, ATP intracellular measurement, XTT test). Several tests based on different principles are often necessary for NPs may sometimes interact with the test principle [22,23]. Nevertheless classic cytotoxicity tests are not early tests since they attest the presence of dead cells. But some deleterious effects may occur before cell death (inflammation, sensibilization, stress oxydant). That is why we used toxicogenomics, meaning the global analysis of gene expression with pangenomic microarrays to obtain an overview of early intracellular events triggered by these surface-treated nanoparticles. With an active toxicant, the gene expression studies, through a large number of altered genes, provide a wealth of information about main altered cellular functions, the mode of action of the substance or the cellular defence mode. These results allow usually to generate new hypotheses about the specific toxicity of concerned substances and thus to carry targeted experiments. We used Scanning Electronic Microscopy (SEM) to visualize cell morphology changes in presence of NPs such as alteration of microvilli, modification of tight junctions or adsorption of aggregates on the cell surface. Finally, we implemented Transmission Electronic Microscopy (TEM) to visualize the internalization of nanoparticles by the cells. This multi pronged approach gives more certainty and coherence to the acquired data.

## Results

### Size, shape and aggregation state of STNPs

While the T-Lite SF® are initially hydrophobic mainly due to the PDMS surface coating, they become hydrophilic when suspended in distilled water, after 48 h of environmental degradation (T-Lite DL) or after 3 h of gastrointestinal degradation (T-Lite DA), because of the loss of PDMS coating. The hydrodynamic diameters (Dh) of these colloidal phases are larger for the T-Lite DA (688 ± 209 nm) compared to the T-Lite DL (237 ± 26 nm) (Table 1). This is consistent with the zeta potentials measured (at pH 8.6, gastrointestinal pH) at the surface of the aged STNPs, which are negative for the T-Lite DL (−25 ± 4 mV) and close to 0 for the T-Lite DA (−2 ± 5 mV) (Table 1). These Dh measured in distilled water were compared to the Dh measured in serum-free culture medium. For both T-Lite DL and T-Lite DA a tendency to aggregate is observed with Dh ranged between 720–1350 nm. These results are consistent with Limbach et al. (2005) [24] showing that oxide NPs with different zeta-potential in ultrapure water, become all homogeneously charged and aggregate once suspended in culture medium.

**Table 1:** Average hydrodynamic diameters and zeta potential of STNPs suspensions

	**In distilled water** ***(pH = 8 ± 0.3)***		**In the serum-free culture medium**
**STNPs**	Hydrodynamic diameters (nm)	Zeta potential (mV) measured	Hydrodynamic diameters (nm)
T-Lite	347 +/− 69	n/a	391+/−15
T-Lite DA	688 +/− 209	−2 +/− 5	723+/−63
T-Lite DL	237 +/− 26	−25 +/− 4	1353+/−231

The STNPs were diluted to 100 μg/mL (T-Lite and T-Lite DA) or 10 μg/mL (T-Lite DL) in distilled water or serum-free culture medium. The measurement was made by DLS and zeta potential measurements.

**Figure 1:**
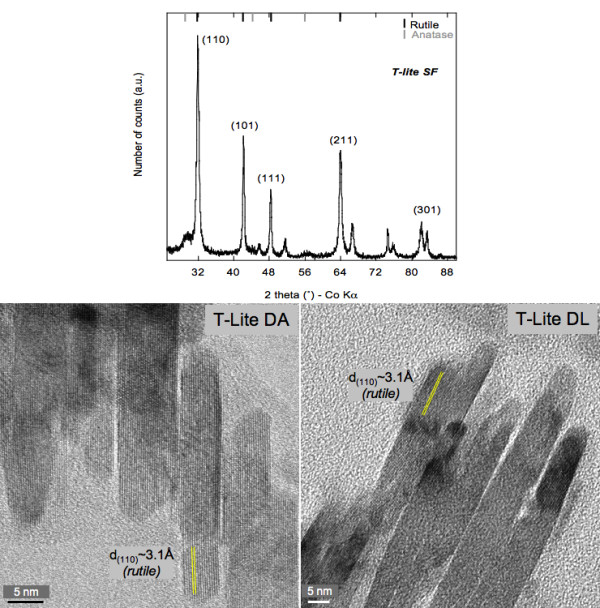
**Shape, size and crystal structure of the T-Lite SF®, T-Lite DA and T-Lite DL.** These high resolution pictures were obtained using the Transmission Electron microscope JEOL 2010 operating at 200 kV. The inter-reticular distances d measured at 3.1 Å for both samples are attributed to the crystalline plane (110) of rutile. The XRD diffractogram was obtained on powder samples analyzed using a PANalytical X’Pert PRO diffractometer with a Co Kα radiation (1.79 Å).

However, these changes in surface properties and physico-chemical behavior in suspension do not affect the crystalline nature, the shape, and the size of the TiO_2_ core of the STNPs. The aging of TiO_2_ STNPs under UV (at neutral pH) or at acid pH, has no effect on the crystalline structure. The inter-reticular distance d(110) (~ 3.1 Å) that characterizes the rutile crystalline phase, is not modified. The shape and size of the rutile core (7 ± 2 nm x 50 ± 10 nm) measured by TEM persist . Such a crystalline structure was confirmed by powder X-ray diffraction (Figure 1).

### Superoxide generation assessment

**Figure 2:**
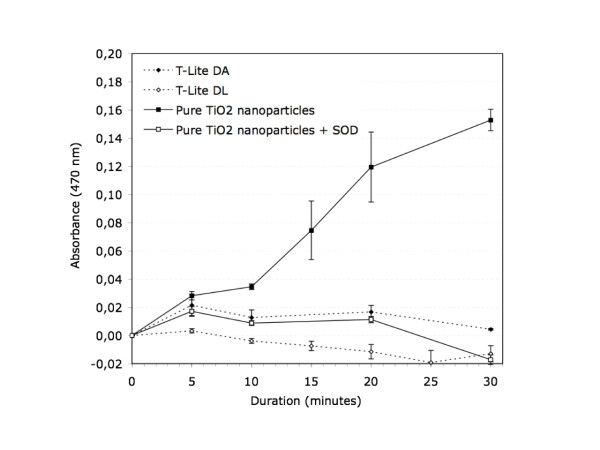
**Superoxide ions generation.** O2-· anions generation by T-Lite DA, T-Lite DL, and pure TiO_2_ nanoparticles measured using the XTT combined or not with the SOD chemical probes. Measurements were performed at 470 nm. Error bars indicate the standard deviation from the mean (n = 3). XTT formazan production = [Abs sample(t)-Abs sample(t0)]-[Abs solvent(t)-Abs solvent(t0)].

The superoxide production by aged T-Lite DA and T-Lite DL was compared to a suspension of surface unmodified TiO_2_ (rutile) nanoparticles under UV light. The physicochemical characterization of these surface unmodified TiO_2_ nanoparticles is available herein [25] and in supporting information [see Additional file 1. The photoactivity of surface unmodified TiO_2_ nanoparticles is obvious (Figure 2), and SOD completely suppressed the XTT-formazan production validating the generation of O_2_^-·^. However no significant amount of O_2_^-·^ is generated in presence of aged T-Lite DA and T-Lite DL in our experimental conditions.

### Cytotoxicity multiparametric tests

**Figure 3:**
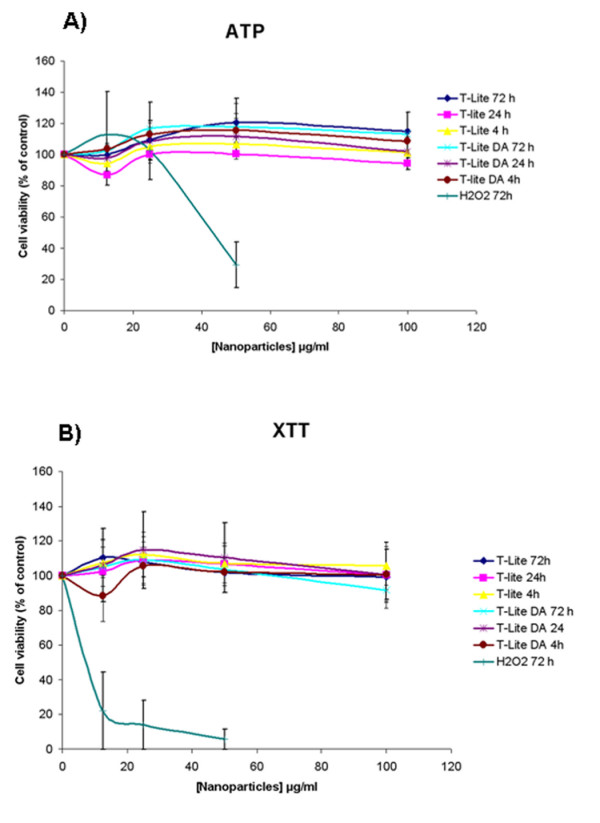
**Viability of Caco-2 cells exposed for 4 h, 24 h or 72 h toTiO**_**2**_**STNPs.** Caco-2 cells were grown in a 96-well plate and differentiated for 21 days. The cells were then exposed for 4 h, 24 h or 72 h at TiO_2_ STNPs concentrations ranging from 10 to 100 μl/mL. **A)** Cell viability was determined by intracellular ATP content ( CellTiter-Glo luminescent cell viability Assay, Promega). **B)** Cell viability was determined by mitochondrial enzyme activity via XTT reagent (In Vitro toxicology assay kit XTT based, Sigma-Aldrich).

**Figure 4:**
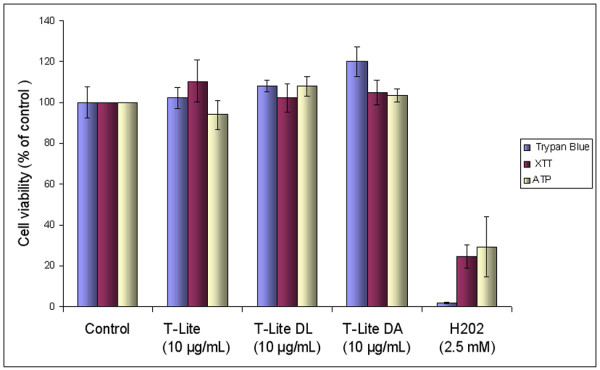
**Multiparametric viability tests of Caco-2 cells exposed for 72 h to 10 μg/mL TiO**_**2**_**STNPs.** Caco-2 cells were grown in 24-well plates and differentiated for 21 days. The cells were exposed for 72 h to various TiO_2_ STNPs at 10 μg/mL. Cell viability was determined by counting the number of viable cells with trypan blue on Cedex (Innovatis), by XTT mitochondrial assay and with ATP measurement. Experimental positive control was obtained by exposing cells to H_2_O_2_ 2.5 mM.

The studies were conducted on the well-established Caco-2 cell line, an intestinal epithelium model known for its relevance in barrier integrity studies, differentiated for 21 days. The integrity of the cell layer testifying to the state of differentiation was determined by measuring the Trans Epithelial Electrical Resistance (TEER) stabilized at 500 Ohms after 21 days. Three different cytotoxicity assays were conducted to test the toxicity of TiO_2_ STNPs to avoid any bias due to possible interference with the test principle, which is common with NPs [22,23]. The first test is an early test of toxicity, because it is based on the measurement of ATP, which assesses the energy state of the cell, even before any damage to membrane integrity occurred. As shown in Figure 3A, the presence of T-Lite and T-Lite DA STNPs did not induce toxic effect on Caco-2 cells after 4 h, 24 h or 72 h exposure or even for concentrations up to 100 μg/mL, using ATP assay. The second test (XTT) is based on the activity of mitochondrial enzymes. Under the same conditions, this assay confirmed the absence of TiO_2_ STNPs toxicity in Caco-2 cells (Figure 3B). The study of T-Lite DL toxicity was limited because of the low concentration of the stock solution (100 μg/mL). As the stock solution must be diluted in the culture medium, the minimum acceptable dilution (so that the medium is sufficiently concentrated) is 1:10. Consequently the maximal concentration of T-Lite DL thus obtained is 10 μg / mL and did not allow testing higher concentrations. Finally, using cell counting, ATP and XTT assays, all T-Lite SNTPs, degraded or not, including TLite-DL, did not show any toxicity (Figure 4) at the concentration of 10 μg/mL after 4, 24 or 72 h, concentration used in gene expression studies thereafter.

### Cell morphology by SEM and localization of STNPs by TEM

**Figure 5:**
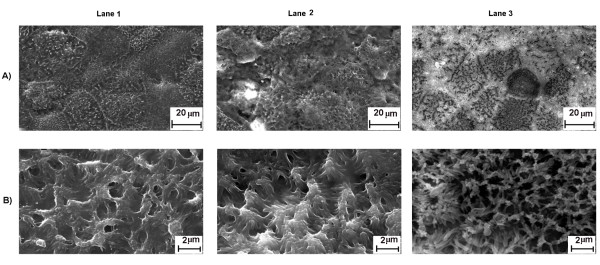
**SEM image of Caco-2 cells exposed for 72 h at 100 μg/mL of STNPs.** Caco-2 cells were grown in a bicameral chamber (PET, pores 1 μm) and differentiated for 21 days. The cells were exposed to STNPs (100 μg/mL). After 72 h incubation, the cells were washed, fixed and dehydrated. They were examined by SEM. **A)** Enlargement X 2000 **B)** Enlargement X 16000. Control, T-Lite and T-Lite DA are in lanes 1, 2, 3 respectively.

The previous cytotoxicity assays did not indicate any toxicity of TiO_2_ STNPs. We also checked cell morphology with SEM. Caco-2 cells exposed for 72 hours to100 μg/mL of STNPs (Figure 5, line A, T-Lite and T-Lite DA, lane 2 and 3 respectively) did not show any alteration in morphology or density compared to control cells (lane 1, line A). The microvilli observed with higher enlargment, were also in good condition (Figure 5, line B). These observations reinforce the absence of toxicity of T-Lite STNPs or their degradation residues.

**Figure 6:**
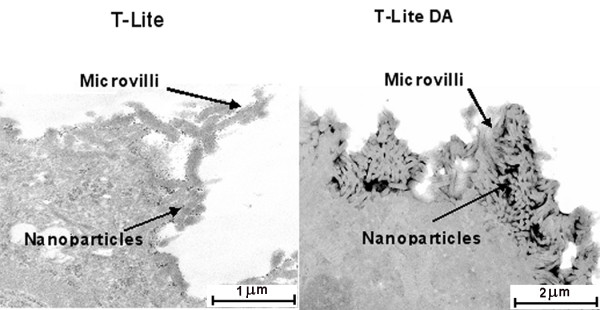
**TEM image of Caco-2 cells exposed to 100 μg/mL of TiO2 STNPs.** Caco-2 cells were cultured for 21 days and differentiated. The cells were exposed for 24 h at 100 μg/mL of TiO_2_ nanoparticles. The cells were washed, fixed, post-fixed, included, cut and stained before being examined by TEM. (Left) T-Lite. (Right) T-Lite DA.

To investigate the ability of TiO_2_ STNPs to cross the intestinal barrier, we performed TEM studies. Light degraded residues could not be analyzed by TEM because the stock solution was too diluted. T-Lite and T-Lite DA were observed at 100 μg/mL. T-Lite STNPs are clearly visible as tiny spots on the surface of the plasma membrane (Figure 6**Left**). More drastically, T-Lite DA SNTPs gather on the cell surface as large aggregates embedded into the microvilli (Figure 6**Right**). This could be explained by an interaction between cells and degraded nanoparticles due to the modification of the protective layer. On the other hand, in both cases, no STNP was visible inside the cell in spite of the multiple ultrathin sections observed. We conclude to no internalization of TiO_2_ STNPs in Caco-2 cells, but that degradation of STNPs favors the sticking to the cell membrane. In addition, STNPs were observed only in the apical side of the cells. This suggests that TiO_2_ STNPs cannot penetrate within the cells and will likely be stopped by the intestinal barrier.

### Gene expression

As no effect on cell viability was observed but increased interaction between cells and degraded TiO_2_ STNPs was suspected, we used gene expression analysis as an ultimate sensitive technique, to detect any change in cell metabolism or regulatory pathways.

**Figure 7:**
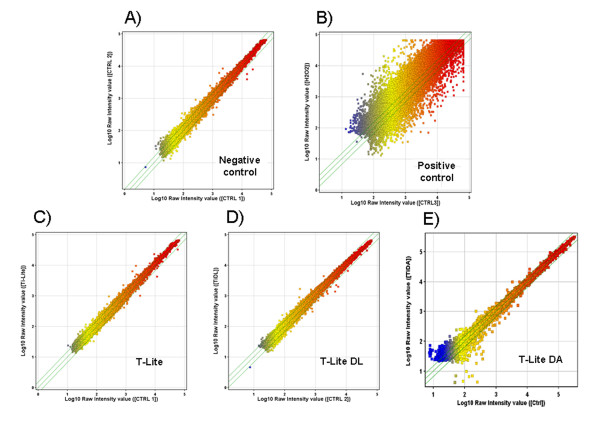
**Microarray scatter plots.** Caco-2 cells were cultured and differentiated for 21 days. The cells were exposed for 72 h at 10 μg/mL of TiO2 STNPs. The scatter plots represent the raw fluorescence intensities of genes signals after hybridization before applying an unpaired t-test statistical analysis and a Benjamini and Hochberg false discovery rate multiple testing correction (n = 4) . **A)** Negative control: unexposed versus unexposed cells (control 2 versus control1). **B)** Positive control: H_2_O_2_ exposed versus unexposed cells (control). **C)** Test: T-Lite STNPs exposed versus unexposed cells (control 1). **D)** Test: T-Lite DL STNPs (UV-degraded) exposed versus unexposed cells (control 2). **E)** Test: T-Lite DA STNPs (acid degraded) exposed versus unexposed cells (control). Figures C) and D) and E) indicate that STNPs exposed and unexposed cells have similar gene expression. The exact number of altered genes after application of statistical tests is reported in Table 2.

Caco-2 cells were exposed to T-Lite STNPs, T-Lite DL and T-Lite DA for 72 h at 10 μg/mL, a very high concentration compared to modeled concentrations in the environment (around 10 μg/L). Oligo microarrays spotted with 41 000 genes were hybridized in quadruplicates with RNA from cells exposed to STNPs or from unexposed cells (for exact design details, see Materials and methods). The scatter plots (Figure 7) represent the raw fluorescence intensities of genes filtered at threshold signal intensity for microarray experiments (n = 4). Moreover, the scatter plots obtained for T-Lite, T-Lite DL or T-Lite DA versus control cells are very similar to the experimental negative control scatter plot obtained from unexposed cells originating from two different cultures (control 2 versus control 1). A positive control scatter plot was obtained with cells exposed to hydrogen peroxide. The exact number of significantly altered genes in all cases is reported in Table 2 and genes names are indicated in additional file 2 [see Additional file 2]. Therefore cells exposed to TiO_2_ STNPs show no significant change in their gene expression.

**Table 2:** Microarray results

**Microarray Analyses**	**Total number of genes**	Number of detected genes*	**Number of genes up- or down- expressed** **(>1.5 fold change )**	**Number of genes** **significantly** **up- or down- expressed** (pvalue < 0.05)**	**% of genes altered out of detected spots**
CTRL 2 vs CTRL 1	41000	23425	970	5	0,021%
Ti-Lite vs CTRL 1	41000	23828	419	1	0,004%
Ti-Lite DL vs CTRL 2	41000	22989	845	2	0,009%
Ti-Lite DA vs CTRL	41000	19928	824	0	0%
H_2_O_2_ vs CTRL	41000	28900	14651	9307	32,2%

Caco-2 cells were cultured and differentiated for 21 days. The cells were exposed for 72 h at 10 μg/mL of TiO_2_ STNPs native or degraded. After mRNA extraction, labeled cDNA (Cy3) was hybridized to Agilent oligomicroarray (41 000 genes). The number of genes detected above a raw intensity threshold (background + 2SD) was compared for each type of STNPs exposed versus unexposed cells (*). From the remaining spots, we selected those with fluorescence ratios (representing STNPs exposed versus unexposed samples) greater than 1.5-fold change cutoff. Then we determined the statistical significance of the changes with pvalue ≤ 0.05 using a student t-test statistical analysis (n = 4) and performing a Benjamini and Hochberg false discovery rate multiple testing correction (**) using Genespring software. At the end of this analysis, we obtained lists of genes which were significantly induced or repressed after exposure to STNPs.

## Discussion

Titanium dioxide nanoparticles represent the most important worldwide production of engineered nanomaterials in term of tons per year (17,000) [26]. In this study, the total TiO_2_ concentrations are deliberately higher than predicted environmental concentrations: 10 to 100 μg/mL versus 0.0007 to 0.016 μg/mL [3]. We took into account a possible increase in the local environment, such as in swimming pool water, spoiled clothing in the workplace, and a child ingesting sunscreen by accident. These residues will likely end up in grey waters and in sludge that can be used as soil fertilizers. Besides, it has been shown that plants can take up and transport nanoparticles as described by Kurepa et al. (2010) [27]and, in this case, they could be ingested by humans.

### Characterization of STNPs

To simulate these exposure pathways, we considered possible degradation by environmental conditions and stomach acidity of the TiO_2_ formulations used in sunscreen. Although the physico-chemical behavior and toxicity of bare TiO_2_ NPs has been documented, nothing has been done about degraded TiO_2_ STNPs that are likely to be encountered in the environment [13]. However, bare TiO_2_ NPs and TiO_2_-based surface-treated nanoparticles used in formulations have different behaviors in aquatic media and different exposure pathways to the organisms. For instance, the surface unmodified TiO_2_ nanoparticles are hydrophilic while the commercialized TiO_2_ STNPs used in sunscreen are hydrophobic due to the PDMS coating. In this study we found that the initially hydrophobic T-Lite SF® becomes hydrophilic after aging in environmental or gastrointestinal conditions. Once introduced in distilled water or in the culture medium they quickly form aggregates. These changes in surface properties are attributed to a desorption and oxidation of the outer organic coating layer made of PMDS as previously demonstrated [15,25]. This change in surface properties can strongly influence toxicity results and depends on the aging conditions. In this regards, we did not use any ultrasonication or dispersant compounds to prevent the aggregation of the TiO_2_ nanoparticles or STNPs *(i)* to mimic real environmental situations and *(ii)* because ultrasonication could modify the surface specificity of STNPs.

Moreover, we investigated the ability of these TiO_2_ STNPs to generate superoxide ions in our experimental conditions. This would highlight a potential toxicity originating from the TiO_2_ phototoxic core. As previously observed, the remaining Al-based layer at the surface of T-Lite DL after alteration prevents the chemical interactions between the Ti atoms of the surface of the TiO_2_ core and the O_2_ and/or H_2_O molecules from the solution. This inhibits the promotion of electron-/hole + of the TiO_2_ core and the ROS generation [15,25]. We found that while surface unmodified TiO_2_ nanoparticles generate O_2_^**.**-^, the T-Lite DA and T-Lite DL do not. Consequently, the Al-based layer at the surface of the TiO_2_ core persists in both T-Lite DL and T-Lite DA and prevents the generation of ROS under UV light.

### Cytotoxicity, cell morphology and localization of STNPs

Through three different toxicity assays, the STNPs tested showed no toxic effect on Caco-2 cells after exposures up to 100 μg/mL over 4 h, 24 h and 72 h. Cell morphology remained intact, confirming the absence of STNPs toxicity. TEM shows broader aggregation of acid-degraded STNPs on the cell membrane, probably due to their larger size, but maybe also to surface charge modifications. But in any case, STNPs are not visible by TEM inside the cytoplasm, meaning that they do not cross the cell membrane. These results obtained at higher concentrations (100 μg/mL) than those encountered in the environment, are consistent with those of previous studies. Our study confirms a previous study by Koeneman and coll. showing that TiO_2_ STNPs does not disturb junction complexes, nor damage the cellular epithelium of Caco-2 cells. Nevertheless these authors indicate that, in their hands, microvilli are disrupted and the levels of intracellular-free calcium increase for all tested concentrations of STNPs under acute conditions but surprisingly not after 10 days at the same concentrations [9]. They suggest that this free calcium elevation could disassemble actin filaments finally absorbed into the cells. In our hands, with described concentrations, we did not observe such changes.

### Gene expression analyses

Transcriptomic analysis, particularly sensitive to the least significant biological change, is a way to highlight genes/proteins which expression is modified by xenobiotics or emergent contaminants such as nanoparticles. For example, in cases of intracellular calcium homeostasis disturbance and free calcium increase, a lot of calcium-binding proteins are overexpressed or underexpressed to restore calcium homeostasis [17]. One might reasonably expect that some families of genes such as cytokines or carriers are affected, for example as a result of an inflammatory response. It turned out that this was not the case. In the current study, transcriptomic results do not show any significant change in gene expression at a concentration of 10 μg/mL, which is certainly higher than modeled environmental concentrations. The number of genes significantly altered by T-Lite, T-Lite DL and T-Lite DA STNPs is negligible (1, 2 and 0 genes respectively), similar to the false positive rate in unexposed cells (5 genes). Genes are described in additional file 2. By comparison, we also performed a positive control by exposing Caco-2 cells to 20 μM hydrogen peroxide for 24 h. H_2_O_2_ is well known for inducing intracellular reactive oxygen species (ROS): using a similar transcriptomic analysis with a fold ratio superior to 1.5, we found 9307 genes altered in Caco-2 cells by H_2_O_2_.

This set of experiments tends to show that STNPs are not harmful to Caco-2 cells after acute exposure. It is also unlikely that TiO_2_ STNPs are noxious for chronic exposure to low doses, since they do not penetrate within these intestinal cells nor modify their gene expression.

## Conclusions

After a detailed physico-chemical characterization of aged surface-treated nanoparticles used in sunscreens (size, shape, zeta potential, surface reactivity), we analyzed their harmfulness in intestinal Caco-2 cell model to mimic a possible contact by oral route. Through three different toxicity assays, the STNPs tested, which have a strong tendency to aggregate in complex media, showed no toxic effect on Caco-2 cells after exposures over 4 h, 24 h and 72 h. Cell morphology remained intact, attested by SEM, and penetration of STNPs was not seen by TEM. Moreover gene expression analysis did not show any significant change versus unexposed cells at a concentration of 10 μg/mL, which is about 1000 times higher than modeled environmental concentrations. It is likely that the Al(OH)_3_ protective layers at the TiO_2_ STNP surface are strong enough to resist to degradation by light or to acidic environment and prevent the generation of ROS. TiO_2_ STNPs, degraded or not, are not harmful to Caco-2 cells and are unlikely to penetrate the body by oral route. The toxicity of nanoparticles and nanomaterials is a difficult issue because it can be considered only on a case by case basis. Nevertheless, we believe that this multi-faceted approach provides robust and reliable results on which nanotechnology's stakeholders can rely on.

## Methods

### STNPs degradation protocols

TiO_2_ STNPs are T-Lite™ SF from BASF, Ludwigshafen, Germany. It consists of TiO_2_ core (10 nm wide and 50 nm length) coated with an Al(OH)_3_ layer and an outer layer of polydimethylsiloxane (PDMS). Two aging protocols were used in this study. First, the T-Lite™ SF were aged in an aqueous solution for 48 h under UV-light to simulate an environmental degradation following the protocol previously published [15]. 400 mg of T-Lite™ SF powder was placed in a wide-mouthed 1 L glass beaker containing 250 mL of ultra pure water and stirred at 690 rpm. Natural sunlight was reproduced using a 400 W sodium discharge lamp, situated 30 cm from the open reactor, and continuously cooled via a connection to air extraction. Whereas the T-Lite™ SF is initially hydrophobic, a stable suspension fraction (non-settled fraction) is formed after 3 hours. After 48 hours this stable suspension is composed of particles with sizes ranging from 300 nm to 6 μm [15]. A 100 mg/L stable stock solution of light-degraded TiO_2_ STNPs (T-Lite DL) was obtained [25]. This stock solution of T-Lite DL was then diluted in culture medium at exposure concentration.

Then a gastrointestinal degradation of the T-Lite SF^TM^ was simulated using a simulated gastric medium (0.2% NaCl, HCl, pH = 1) for 3 h at 37°C ([STNPs] = 5 mg/mL). The solution was then neutralized by adding NaHCO_3_[16]. The stock solution obtained (T-Lite DA) was then diluted in the culture medium at the exposure concentration.

### Physico-chemical characterization of the aged STNPs

These aged suspensions (T-Lite DL and T-Lite DA) were physico-chemically characterized in term of shape, size, crystal structure, hydrodynamic diameter, zeta potential, and superoxide generation. Hydrodynamic diameter were measured by dynamic light scattering (3 run, n = 3) in pure water and in the serum-free culture medium using the Zetasizer nano ZS (Malvern instruments Ltd, Worcestershire, UK). The zeta potential was measured at the end of the aging to better understand the evolution of the STNPs surface chemistry. The apparatus used was a Malvern Zetasizer Nano Z from Malvern Instruments (Malvern, UK), working in mixed field mode. The size of the nanoparticles, their shape and crystal structure was assessed using a Transmission Electron Microscope JEOL 2010 operating at 200 kV. Samples were prepared by evaporating a droplet of the STNPs suspensions on a carbon coated copper grid at room temperature.

### Superoxide generation assessment

The O_2_^-·^ production by the surface of aged STNPs was compared with a suspension of pure TiO_2_ nanoparticles [28]. The reduction of 2,3- bis(2-methoxy-4-nitro-5-sulfophenyl)-2 H-tetrazolium-5-carboxanilide (XTT) allows for specific targeting and measurement of superoxide anions [29,30] when it is combined with O_2_^-·^, a quencher of superoxide which cells utilize for protection [31]. XTT reduction by O_2_^-·^ results in the formation of XTT-formazan producing an absorption peak at 470 nm that can be used to quantify the relative amount of superoxide present. Experiments were performed on three individual suspensions: TiO_2_ nanoparticles [28], the T-Lite DA and the T-Lite DL suspended in pure water (pH 7) at a concentration of 10 mg/L. 10 mL of each suspension was magnetically stirred and exposed to UV light (Philips TL-D 15 W/08) within a UV box for up to 30 min in the presence of XTT and SOD at a concentration of 100 μM and 25 U/mL, respectively. Measurements were performed on a Cintra 10 spectrometer.

### Cell culture

Caco-2 cells were cultured in Eagle's Minimum Essential Medium (ATCC, Manassas, VA, USA) supplemented with 10% FCS (LGC Standards, Middlesex, UK) and penicillin / streptomycin (100 μg/mL) in a humidified incubator at 37°C and 5% CO2. Cells were used between passages 20 to 40. Cells were passed weekly at a seeding concentration of 6.10^3^ cells/cm^2^ and the medium was changed 3 times per week. For experiments, the cells were seeded in the medium at 5x10^4^ cells/cm^2^ and allowed to differentiate for 21 days. The permeability of the cell layer and the state of differentiation were determined by measuring the Trans-Epithelial Electrical Resistance (TEER) with an electrode on an STX2 EvomX device (World Precision Instruments, Inc, Sarasota, Florida, USA).

### Cytotoxicity multiparametric tests

#### ATP test

Caco-2 cells were grown in a 96-well plate and differentiated for 21 days. Cells were exposed for 4 h, 24 h or 72 h at various concentrations of altered TiO_2_ STNPs (10 to 100 μg/mL, 100 μl per well) (T-Lite DL and T-Lite DA). Cell viability was determined by the ATP test as specified by the supplier (CellTiter-Glo luminescent cell viability Assay, Promega). Briefly, 100 μl of kit reagent were added per well, the plate was shaken for 10 min at RT before measuring bioluminescence (LUMIstar Galaxy, BMG). Hydrogen peroxide (2.5 mM, 1.25 mM and 0.625 mM) was used as positive control.

#### XTT test

Caco-2 cells were grown in a 96-well plate and differentiated for 21 days. The cells were exposed for 4 h, 24 h or 72 h at various concentrations of altered TiO_2_ STNPs (10 to 100 μg/mL, 100 μL per well). Cell viability was determined by the XTT test as specified by the supplier (In Vitro toxicology assay kit XTT based, Sigma-Aldrich). Briefly, 20 μL of kit reagent were added per well, the plate was incubated for 2 h at 37°C before reading absorbance at 450 nm and 690 nm (Multiscan Spectrum, Thermo Electron Corporation). Hydrogen peroxide (2.5 mM, 1.25 mM and 0.625 mM) was used as positive control.

#### Trypan blue test

Caco-2 cells were grown in a 96-well plate and differentiated for 21 days. The cells were exposed for 72 h at 100 μg/mL (T-Lite and T-Lite DA STNPs) and 10 μg/mL for T-Lite DL. After STNPs exposure, the cells were washed 3 times with PBS, collected by trypsinization, washed twice with PBS, and resuspended in PBS before being counted in a cytometer (Cedex) in the presence of trypan blue. The results are expressed as % of viable cells in the sample over the control (unexposed cells). Hydrogen peroxide (2.5 mM) was used as positive control.

### Scanning electron microscopy (SEM ) and transmission electron microscopy (TEM)

Caco-2 cells were grown in a bicameral chamber (membrane PET, 1 μm pores, VWR) and differentiated for 21 days. The cells were exposed to degraded TiO_2_ STNPs (100 μg/mL). After 72 h, the cells were washed 3 times with PBS, fixed with glutaraldehyde 5% in 0.1 M cacodylate for 1 h at 4°C, then washed again twice with distilled water and dehydrated by graded ethanol baths (35, 70, 85, 95 and 100%). Finally, the cells were dehydrated in HMDS (SPI-Chem^TM^) before examination by SEM.

For TEM experiments, Caco-2 cells were grown in a 60 mm diameter Petri dish and differentiated for 21 days. The cells were exposed for 24 h at 100 μg/mL T-Lite or T-Lite DA (3 mL/dish). They were then washed once with medium and once with PBS. The cells were fixed in 2.5% glutaraldehyde in 0.1 M cacodylate for 30 min at 4°C. They were washed 3 times in cacodylate, post-fixed with osmium acid, embedded in epoxy resin, cut and stained before being observed in TEM.

### Microarrays and gene expression analysis

Caco-2 cells were grown in 6-well plates and differentiated for 21 days. The cells were exposed for 72 h to 10 μg/mL TiO_2_ STNPs (3 mL/well) in duplicates. Control duplicates were achieved in the vehicle. Each condition of exposure to nanoparticles had its own control, i.e. unexposed cells (Control 1 for T-Lite, Control 2 for T-Lite DL and Control for T-Lite DA). Additionally, the cells were also exposed to hydrogen peroxide for 24 h at 20 μM as a positive control. The cells were washed extensively to avoid co-extraction of nucleic acids with STNPs adsorbed on the cell surface, collected with trypsin and washed with PBS. The cells were centrifuged and RNA extracted using the Rneasy kit (Qiagen). The RNAs were quantified with the Nanodrop 1000 and their quality analyzed on an Agilent Bioanalyzer 2100. The RNAs were amplified and labeled with cyanine-3 fluorophore using a QuickAmp kit (Agilent) according to the supplier’s protocol. The efficiency of fluorescent labeling was controlled by UV spectroscopy (Nanodrop 1000) before hybridization on Agilent oligo microarrays (Human V2 4X 44 K) in technical duplicates. The microarrays were scanned with a GenePix 4000B (Axon Instrument Inc., Forster City, CA) in one-color mode at 532 nm, PMT 450 nm and 5 μm resolution. Each condition of exposure to nanoparticles as well as controls led to four hybridizations (two biological replicates and two technical replicates), which were needed to achieve sufficient statistical power (n = 4).

In this experimental design, five analyses were conducted: i) STNPs T-Lite exposed cells versus unexposed cells (named control 1), ii) T-Lite DL STNPs exposed versus unexposed cells (named control 2) iii) T-Lite DA STNPs exposed versus unexposed cells (named control), iiii) control 2 versus control 1 as experimental negative control and iiiii) hydrogen peroxide exposed cells versus unexposed cells (named control) as experimental positive control. For each analysis, eight raw fluorescence data files with gpr extension, obtained after scanning and corresponding to four exposed cultures and four control cultures, were submitted to GeneSpring software GX11 (Agilent Technologies) as follows. Concerning the statistics methodology, we used a widespread method for determining the significance change of gene expression [32]. The raw data were first normalized using the percentile shift 75 normalization method. The normalized data were then filtered on the basis of spots presence on 100% of the slides in one of two conditions (STNPs exposed or control). Only spots detected with at least 70% of their pixels above the threshold intensity signal (set to the median background plus two standard deviations) were selected. From the remaining spots, we selected those with fluorescence ratios (representing STNPs exposed samples versus unexposed samples) greater than 1.5-fold change cutoff, then we determined the statistical significance of the changes with pvalue ≤ 0.05 using a student t-test statistical analysis on Genespring software and performing a Benjamini and Hochberg false discovery rate multiple testing correction. At the end of this rigorous analysis, we obtained lists of genes which are significantly induced or repressed after exposure to STNPs.

## Abbreviations

NPs: Nanoparticles; STNPs: Surface-treated nanoparticles; T-Lite DL is T: Lite STNP degraded by light exposure; T-Lite DA is T: Lite STNP degraded by acidic treatment with SGF; SFG: Simulated gastric fluid; TEER: Trans-Epithelial Electric Resistance; SEM: Scanning Electronic Microscopy; TEM: Transmission Electronic Microscopy; PMT: Photomultiplier; FCS: Fetal calf serum; PEG: Polyethylene glycol; PBS: Phosphate buffer saline; ROS: Reactive oxygen species; PMDS: Polydimethylsiloxane; HMDS: Hexamethyldisilazane; d inter reticular distance: Dh hydrodynamic diameters.

## Competing interests

The authors declare that they have no competing interests.

## Authors' contributions

MF carried out the cell cultures, viability studies, size determination, participated in the microarray experiments and drafted the manuscript. GS performed RNA extraction and labeling and microarray experiments. FB carried out the statistical analysis and bioinformatics studies. MA performed the physico-chemical characterization and ROS determination. JR coordinated the different teams taking part in the whole project and provided the STNPs. OP conceived the study, participated in its design, coordinated the experiments and wrote the final manuscript. All authors read and approved the final manuscript.

## Supplementary Material

Additional file 1 **(physicochemical characterization of surface unmodified TiO**_2_** nanoparticles).** Click here for file

Additional file 2 (list of deregulated genes). Click here for file
