# Supplementary material for: Intestinal toxicity evaluation of TiO2 degraded surface-treated nanoparticles: a combined physico-chemical and toxicogenomics approach in caco-2 cells
Source: Part Fibre Toxicol. 2012 May 31;9:18. doi: 10.1186/1743-8977-9-18 (PMC3583216; doi:10.1186/1743-8977-9-18)
Supplement: Additional file 1 — (physicochemical characterization of surface unmodified TiO2 nanoparticles). [file 1743-8977-9-18-S1.pdf]

Additional file 1

The "surface unmodified TiO2" that refers to uncoated rutile nanoparticles. They have been prepared by a simple route involving the hydrolysis of $TiCl_4$ solution in hydrochloric acid (Pottier, A. et al. *Journal of Materials Chemistry* **2001** *11*(4) 1116-1121). TEM micrograph of thus prepared particles (**Figure S1**) shows a rodlike morphology. Nanorods are quite polydispersed in size with an average length *L* of 120 nm and average diameter *D* of 20 nm (aspect ratio *L/D=6)*. Details have been added in the manuscript.

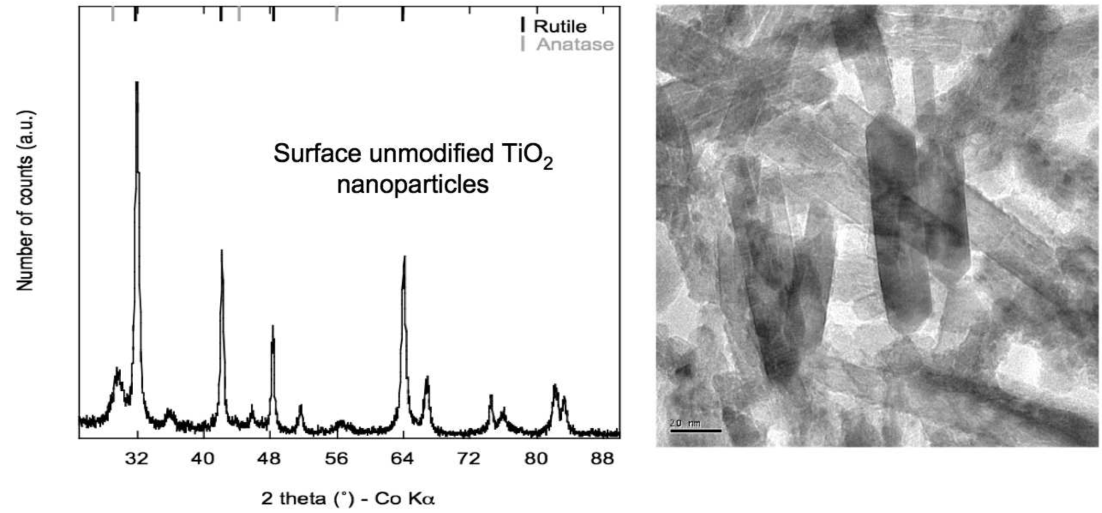

Figure S1. Physico-chemical characterization of the surface unmodified TiO_2 nanoparticles. (right) TEM image of rutile nanorods prepared by thermolysis of $TiCl_4$ suspension. Rutile nanorods were elongated along the [101] direction, (left) X-ray diffraction pattern of the pure rutile nanorods.
