# Supplementary material for: Intestinal toxicity evaluation of TiO2 degraded surface-treated nanoparticles: a combined physico-chemical and toxicogenomics approach in caco-2 cells
Source: Part Fibre Toxicol. 2012 May 31;9:18. doi: 10.1186/1743-8977-9-18 (PMC3583216; doi:10.1186/1743-8977-9-18)
Supplement: Additional file 2 — (list of deregulated genes). [file 1743-8977-9-18-S2.doc]

Additional file 2

**Legend: List of over- or under-expressed genes**

For control, these genes have no reason to be up or down regulated and constitute false positive results. For T-Lite, one sole gene (COBW) is over-expressed; it is a clone without any known function. For T-Lite DL, FAM64A and S100 A4 are up- and down-regulated respectively. FAP64A has no identified function and S100A4 codes for a calcium-binding protein. These genes are not really representative of a toxic effect neither by their small number, nor by their low fold change.
